# Difficulty accessing supervised consumption services during the COVID-19 pandemic among people who use drugs in Vancouver, Canada

**DOI:** 10.1186/s12954-022-00712-7

**Published:** 2022-11-18

**Authors:** Rachel Cassie, Kanna Hayashi, Kora DeBeck, M.-J. Milloy, Zishan Cui, Carol Strike, Jeff West, Mary Clare Kennedy

**Affiliations:** 1 School of Public Health, 3-300 Edmonton Clinic Health Academy, University of Alberta, 11405 87 Ave., Edmonton, AB T6G 1C9 Canada; 2 British Columbia Centre on Substance Use, 400-1045 Howe Street, Vancouver, BC V6Z 2A9 Canada; 3 Faculty of Health Sciences, Simon Fraser University, Blusson Hall, Room 11300, 8888 University Drive, Burnaby, BC V5A 1S6 Canada; 4 School of Public Policy, Simon Fraser University, 8888 University Drive, Burnaby, BC V5A 1S6 Canada; 5 Department of Medicine, St. Paul’s Hospital, University of British Columbia, 608-1081 Burrard Street, Vancouver, BC V6Z 1Y6 Canada; 6 Dalla Lana School of Public Health, University of Toronto, 155 College St., Toronto, ON M5T 3M7 Canada; 7 La Ka Shing Knowledge Institute, St. Michael’s Hospital, 209 Victoria St., Toronto, ON M5B 1T8 Canada; 8 Vancouver Coastal Health, 828 W 10th Ave., Vancouver, BC V5Z 1L8 Canada; 9 School of Social Work, University of British Columbia - Okanagan, 1147 Research Road (ARTS Building), Kelowna, BC V1V 1V7 Canada

**Keywords:** Supervised consumption site, Overdose prevention site, COVID-19, Overdose crisis, Illicit drug use, Addiction

## Abstract

**Background:**

The overdose crisis in Canada has worsened since the emergence of the COVID-19 pandemic. Although this trend is thought to be driven in part by closures or reduced capacity of supervised consumption services (SCS), little is known about the factors that may impede access to such services during the COVID-19 pandemic among people who use drugs. This study sought to characterize the prevalence and correlates of having difficulty accessing SCS during the COVID-19 pandemic among people who use drugs in Vancouver, Canada.

**Methods:**

Cross-sectional data from two open prospective cohorts of people who use drugs were collected via phone or videoconferencing interviews conducted between July 2020 and November 2020. Multivariable logistic regression analyses were used to examine factors associated with experiencing difficulty accessing SCS.

**Results:**

Among the 428 people who use drugs who participated in the study, 223 (54.7%) self-identified as men and the median age was 51 years (1st to 3rd quartile: 42–58). A total of 58 (13.6%) participants reported experiencing difficulty accessing SCS. In a multivariable analysis, factors positively associated with difficulty accessing SCS included daily crystal methamphetamine use (Adjusted odds ratio [AOR] = 2.60; 95% confidence interval [CI] 1.28–5.30), active injection drug use (AOR = 4.06; 95% CI 1.38–11.90), recent non-fatal overdose (AOR = 2.45; 95% CI 1.24–4.85), and unstable housing (AOR = 2.14; 95% CI 1.08–4.23). Age was inversely associated with the outcome (AOR = 0.96; 95% CI 0.93–0.99) in multivariable analyses. The most commonly reported reasons for experiencing difficulty accessing SCS were: COVID-19-related site closure or shortened hours (42.9%) and having to wait too long to use a site (39.3%).

**Conclusions:**

We found that people who use drugs with markers of structural vulnerability and drug-related risk were more likely to experience difficulty accessing SCS during the COVID-19 pandemic. These findings point to the need for strategies to support access to such services as part of pandemic response efforts.

## Background

Fuelled in large part by the proliferation of illicitly manufactured fentanyl and fentanyl analogues in the unregulated drug market, the overdose crisis continues to have devastating impacts in the USA and Canada [1, 2], especially in the province of British Columbia (BC) [3]. In 2016, the BC provincial government declared the overdose crisis a public health emergency [3]. Of Canada’s provinces, BC experienced the highest fatal overdose rate in 2020 (35 deaths per 100,000 population), with fentanyl detected in 86% of these fatalities [4].

Several interventions have been implemented in response to the overdose crisis, including the scale-up of supervised consumption services (SCS) in Canada [5–7]. SCS provide safe and regulated spaces in which people can consume drugs, including unregulated drugs and pharmaceutical drugs, under the supervision of trained staff [5–13]. These services provide clients with sterile drug supplies, education of safer using techniques, and rapid response in the event of an overdose, including administration of supplemental oxygenation and naloxone [5–13]. SCS also often provide other low-barrier services on site (e.g. nursing care, drug checking) and referrals to external healthcare services such as hospitals, primary care clinics, detoxification services, and housing [7, 9–11, 13]. Since 2016, the number of federally sanctioned SCS (i.e. sites with an exemption from Health Canada under the Controlled Drugs and Substances Act) operating in Canada has increased from one to 39 sites (as of November 2022) [14]. In addition, approximately 40 low-barrier SCS, referred to as overdose prevention sites (OPS), opened in Canada between December 2016 and December 2021 [15, 16]. OPS operate outside of federal approval processes and governing regulations and were first sanctioned when the BC Minister of Health issued a ministerial order in December 2016 that directed health authorities in the province to immediately establish and fund these sites [7]. As of December 2021, there were twelve SCS operating in the Greater Vancouver region of BC, including four federally sanctioned SCS and eight community-based OPS [14, 17]. Additionally, a number of housing-based OPS have been established in non-profit organization-operated housing buildings in Vancouver, primarily in single-room occupancy housing buildings [18–20]. Housing-based OPS are often available for use by tenants only, although some are also accessible to guests [18–20].

The overdose crisis has worsened with the emergence of COVID-19, resulting in people who use drugs experiencing converging public health crises [21, 22]. Between April 2020 and March 2021, Canada experienced an 88% increase in the number of apparent opioid toxicity deaths compared to the same time period in the previous year [2]. The heightened overdose crisis is likely related to numerous COVID-19 response measures including border closures that have increased the toxicity of the unregulated drug market by disrupting drug supply chains, and physical distancing measures that may have increased the prevalence of people using alone [21–25]. Additionally, the rise in overdose deaths during the COVID era could be due in part to interrupted access to key harm reduction services, including SCS, which have been shown to be effective in reducing risk of overdose-related harms [5, 21, 22, 26]. With the emergence of COVID-19, many SCS in Canada were forced to close or temporarily restrict access to facilitate physical distancing [21, 22]. For example, in Vancouver, one SCS closed because the site was unable to ensure physical distancing, while other SCS operated at reduced capacity in the first several months of the pandemic [27]. Further, SCS in Vancouver and other settings in Canada have implemented additional COVID-19 response measures that could potentially have impeded service accessibility and engagement, including temporarily modifying overdose response procedures, such as requiring service users to leave the site when providing artificial respirations, and suspending some ancillary services [27, 28].

Despite these changes to SCS operations since the emergence of COVID-19, the impacts of the COVID-19 pandemic and related response measures on SCS use among people who use drugs have not yet been well examined. In particular, little is known about individual and social–structural factors associated with experiencing barriers in accessing SCS during the COVID-19 pandemic, or reasons for experiencing such barriers. Therefore, we sought to characterize the prevalence and correlates of having difficulty accessing SCS (including federally sanctioned SCS and/or OPS) during the COVID-19 pandemic among participants in two community-recruited cohorts of people who use drugs in Vancouver, Canada. We also descriptively examined self-reported reasons for experiencing difficulty accessing SCS among this group.

## Methods

Data were drawn from two open prospective cohort studies of people who use drugs in Vancouver, Canada: the Vancouver Injection Drug Users Study (VIDUS) and the AIDS Care Cohort to evaluate Exposure to Survival Services (ACCESS). These cohorts have been described in detail in previous publications [29]. Briefly, since May 2005, participants have been recruited through community-based methods including self-referral and street outreach. VIDUS is a cohort of HIV-negative adult people who inject drugs who report unregulated drug injection at least once in the month before enrolment. ACCESS is a cohort of adults living with HIV and who have used unregulated drugs besides or in addition to cannabis in the previous month at enrolment. VIDUS participants who seroconvert to HIV after enrolment are transferred to the ACCESS cohort. All participants provide written informed consent. The studies utilize harmonized data collection and follow-up procedures to facilitate pooled analyses.

At baseline and semi-annually after enrolment, study participants complete an interviewer-administered questionnaire and provide blood samples for serological testing or HIV clinical monitoring, as appropriate. The questionnaire collects information related to socio-demographic characteristics, substance use and other behaviours, social–structural exposures, and healthcare services engagement. Participants receive a stipend of $40 CAD at each study visit. These studies have ethics approval from Providence Health Care/University of British Columbia’s Research Ethics Board.

In March 2020, in-person data collection activities for VIDUS and ACCESS, including study visits to complete questionnaires and provide blood samples for serological testing, were suspended due to the COVID-19 pandemic. Between March and July 2020, study measures to reduce the risk of COVID-19 transmission were developed and the study questionnaire was revised to include questionnaire items related to the COVID-19 pandemic. In July 2020, data collection resumed. Study interviews were conducted exclusively via telephone or videoconferencing rather than via in-person visits. Participants were notified using telephone or social media that they were due for a follow-up interview and that these interviews would occur remotely. Interested participants were given the option of completing a study interview via telephone or videoconferencing technology. Those who did not have access to a telephone could use a study-owned pre-paid cell phone to conduct the interview. Additionally, a space was provided for participants who did not have access to a private space to complete an interview. Participants who had access to a bank account received their honorarium via e-transfer. Arrangements were made to allow those without access to a bank account to pick up cash honoraria in person.

The present cross-sectional analyses were restricted to VIDUS and ACCESS participants who completed a remote study interview between July 2020 and November 2020 in which they reported having used drugs (including use of unregulated drugs and/or prescription drugs used for non-medical purposes) in the last six months. The primary outcome for this study was difficulty accessing SCS during the COVID-19 pandemic, which was determined by asking participants: “In the last six months, was there a time when you wanted to use these sites [i.e. supervised consumption or overdose prevention sites] but were unable to?” (yes vs. no). Explanatory variables were selected based on previous studies that examined the utilization of SCS among people who use drugs [30–32], and a priori hypothesized associations. These variables included the following sociodemographic variables: age (per year increase); self-identified gender (men vs. women or non-binary); ancestry (white vs. Black, Indigenous, and people of colour); education (≥ high school vs. < high school); employment (yes vs. no); and residence in Downtown Eastside (yes vs. no). The Downtown Eastside is the neighbourhood where most SCS in Vancouver are located that is characterized by a large open drug scene and high levels of poverty, homelessness and overdose deaths [33, 34]. We also considered a range of drug use variables including ≥ daily use of unregulated opioids (i.e. heroin, fentanyl, or unspecified ‘down,’ which refers to a combination of heroin and fentanyl [35]) (yes vs. no); ≥ daily non-medical prescription opioid use (yes vs. no); ≥ daily cocaine use (yes vs. no); ≥ daily crystal methamphetamine use (yes vs. no); ≥ daily crack cocaine use (yes vs. no); benzodiazepine use (yes vs. no); speedball (i.e. mixture of unregulated opioids and cocaine) use (yes vs. no); goofball (i.e. mixture of unregulated opioids and methamphetamine) use (yes vs. no); any injection drug use (yes vs. no); used drugs alone (yes vs. no); syringe/drug use equipment sharing (yes vs. no); non-fatal overdose (yes vs. no); and perceived drug quality since COVID-19 pandemic (worse vs. same or better compared to before COVID. Additionally, we considered health-related and social–structural exposure variables including participation in any form of addiction treatment (yes vs. no); experienced physical violence (yes vs. no); unstable housing (yes vs. no); sex work involvement (yes vs. no); incarceration (yes vs. no); cohort/HIV status (VIDUS vs. ACCESS); believe to have ever been infected with COVID-19 (yes vs. no). Consistent with the past work [36], unstable housing was defined as living in a single-room occupancy hotel, shelter, recovery or transition house, jail, on the street, or having no fixed address. All drug use, behavioural, and experiential variables refer to the last 6 months prior to interview date unless otherwise indicated. All drug use variables refer to perceived use, which could include intentional and/or unintentional use.

Bivariable analyses were used to assess the association between each explanatory variable and difficulty accessing SCS. Categorical explanatory variables were analysed using Pearson’s Chi-squared test or Fisher’s exact test when expected cell counts were less than or equal to five. Continuous variables were assessed using the Mann–Whitney U test. Unadjusted odds ratios were estimated using bivariable logistic regression. Next, a multivariable logistic regression model was built using an a priori-defined statistical protocol based on appraisal of two criteria: the Akaike information criterion (AIC) and Type III *p* values. This procedure selects models based on an optimization of best explanatory model with best model fit [37]. The initial model included all variables with a *p* value < 0.50 in bivariable analyses. After noting the model AIC, the variable with the largest type III *p *value was removed to create a reduced model. We proceeded with this iterative process of noting the AIC and removing the variable with the largest *p* value, until the model had no explanatory variables remaining. The multivariable model with the smallest AIC during this process was chosen as the final model. The amount of missing data was minimal (3.7% overall), and therefore, missing data were removed from the multivariable model.

As a sub-analysis, we produced descriptive statistics for responses to the question: “If yes, why were you unable to use these sites?” among participants who reported having difficulty accessing SCS. Response options (which were not read aloud to participants) included: the site closed or had shortened hours due to COVID-19; have to wait too long to inject; too hectic; moved away from these sites; too many rules and restrictions; police loitering around the sites; don’t feel safe going there; fear of getting COVID-19; closed for reason other than COVID-19; other (specify). Participants could provide more than one response. All statistical analyses were performed with SAS version 9.4 (SAS Institute Inc., Cary, NC, USA), and all *p *values were two-sided.

## Results

Among 428 people who use drugs included in the study, the median age was 51 years (1st to 3rd quartile = 42–58), 223 (54.7%) self-identified as men, and 222 (54.7%) self-reported their ancestry as white. In total, 58 (13.6%) reported experiencing difficulty accessing SCS in the last six months.

In bivariable analyses (Table 1), variables that were significantly and positively associated with difficulty accessing SCS were: ≥ daily unregulated opioid use (odds ratio [OR] = 2.43; 95% confidence interval [CI] = 1.35–4.36), ≥ daily crystal methamphetamine use (OR = 2.90; 95% CI 1.56–5.40), benzodiazepine use (OR = 3.78; 95% CI 1.22–11.72), injection drug use (OR = 6.17; 95% CI 2.18–17.43), participation in addiction treatment (OR = 2.33; 95% CI 1.11–4.91), recent non-fatal overdose (OR = 2.66; 95% CI 1.43–4.93), experienced physical violence (OR = 2.40; 95% CI 1.24–4.65), unstable housing (OR = 2.58; 95% CI 1.39–4.82), and perceiving worse drug quality since COVID-19 (OR = 2.12; 95% CI 1.19–3.77). Age (OR = 0.93; 95% CI 0.91–0.96) and self-identifying as a man (OR = 0.50; 95% CI 0.28–0.88) were inversely associated with the outcome in bivariable analyses. In a multivariable analysis (Table 2), variables that remained significantly and positively associated with difficulty accessing SCS included ≥ daily crystal methamphetamine use (adjusted odds ratio [AOR] = 2.60; 95% CI 1.28–5.30), active injection drug use (AOR = 4.06; 95% CI 1.38–11.90), recent non-fatal overdose (AOR = 2.45; 95% CI 1.24–4.85), and unstable housing (AOR = 2.14 95% CI 1.08–4.23). Age was inversely associated with the outcome (AOR = 0.96; 95% CI 0.93–0.99) in the multivariable analysis.

**Table 1:** Bivariable analyses of factors associated with difficulty accessing supervised consumption services or overdose prevention sites during the COVID-19 pandemic among 428 people who use drugs in Vancouver, Canada (2020)

Characteristic	Difficulty accessing SCSs or OPSs in last six months		Odds ratio (95% CI)	*p *value
	Yes, *n* (%) *n* = 58 (13.6%)	No, n (%) *n = 370* (86.4%)		
*Age (per year increase)*				
Median (IQR^†^)	44 (35–52)	52 (44–58)	0.93 (0.91–0.96)	< 0.001
*Self-Identified Gender* *Gender*				
Men	23 (39.7)	210 (56.8)	0.50 (0.28–0.88)	0.015
Women/non-binary	35 (60.3)	160 (43.2)		
*Ancestry*				
White	26 (44.8)	208 (56.5)	0.63 (0.36—1.09)	0.096
Black, Indigenous, and people of colour	22 (55.2)	160 (43.5)		
*Education*				
High school or greater	27 (46.6)	179 (49.2)	0.90 (0.52–1.57)	0.710
Less than high school	31 (53.5)	185 (50.8)		
*Employment*				
Yes	21 (36.2)	124 (33.5)	1.13 (0.63–2.01)	0.687
No	37 (63.8)	246 (66.5)		
Downtown Eastside residence^*^				
Yes	39 (67.2)	219 (59.2)	1.42 (0.79–2.54)	0.244
No	19 (32.8)	151 (40.8)		
*≥ Daily unregulated* opioid use*				
Yes	39 (67.2)	169 (45.8)	2.43 (1.35–4.36)	0.002
No	19 (32.8)	200 (54.2)		
≥ Daily non-medical prescription opioid use*				
Yes	1 (1.8)	8 (2.2)	0.81 (0.10–6.58)	1.000^Ψ^
No	56 (98.3)	362 (97.8)		
≥ Daily cocaine use*				
Yes	2 (3.5)	11 (3.0)	1.16 (0.25–5.38)	0.693^Ψ^
No	56 (96.6)	358 (97.0)		
≥ Daily crystal methamphetamine use*				
Yes	19 (32.8)	53 (14.4)	2.90 (1.56–5.40)	0.001
No	39 (67.2)	316 (85.6)		
≥ Daily crack use*				
Yes	6 (10.3)	54 (14.6)	0.68 (0.28–1.65)	0.386
No	52 (89.7)	316 (85.4)		
Benzodiazepine use*				
Yes	5 (8.6)	9 (2.4)	3.78 (1.22–11.72)	0.029^Ψ^
No	53 (91.4)	361 (97.6)		
Speedball use*				
Yes	0 (0.0)	20 (5.4)	N/A φ	0.091^Ψ^
No	58 (100)	350 (94.6)		
Goofball use*				
Yes	14 (24.1)	53 (14.3)	1.90 (0.98–3.71)	0.056
No	44 (75.9)	317 (85.7)		
Injection drug use*				
Yes	54 (93.1)	254 (68.7)	6.17 (2.18–17.43)	0.001
No	4 (6.9)	116 (31.4)		
Used drugs alone*				
Yes	41 (78.9)	193 (80.8)	0.89 (0.42–1.86)	0.754
No	11 (21.2)	46 (19.3)		
Participation in addiction treatment*				
Yes	49 (84.5)	257 (70.0)	2.33 (1.11–4.91)	0.023
No	9 (15.5)	110 (30.0)		
Non-fatal overdose*				
Yes	19 (32.8)	57 (15.5)	2.66 (1.43–4.93)	0.001
No	39 (67.2)	311 (84.5)		
Experienced physical violence*				
Yes	15 (25.9)	47 (12.7)	2.40 (1.24–4.65)	0.008
No	43 (74.1)	323 (87.3)		
Syringe/drug use equipment sharing*				
Yes	17 (30.4)	82 (23.2)	1.44 (0.77–2.68)	0.247
No	39 (69.6)	271 (76.8)		
Unstable housing*				
Yes	43 (74.1)	193 (52.6)	2.58 (1.39–4.82)	0.002
No	15 (25.9)	174 (47.4)		
Sex work involvement*				
Yes	9 (15.8)	38 (10.4)	1.61 (0.73–3.54)	0.230
No	48 (84.2)	327 (89.6)		
Incarceration*				
Yes	2 (3.5)	9 (2.5)	1.43 (0.30–6.77)	0.651^Ψ^
No	56 (96.6)	359 (97.6)		
*Cohort/HIV status*				
Yes	38 (65.5)	199 (53.8)	1.63 (0.92–2.91)	0.095
No	20 (34.5)	171 (46.2)		
*Believe been infected with COVID-19*				
Yes	4 (7.1)	23 (6.3)	1.14 (0.38–3.44)	0.771
No	52 (92.9)	342 (93.7)		
*Perceived drug quality since COVID-19 pandemic*				
Worse	34 (60.7)	153 (42.2)	2.12 (1.19–3.77)	0.009
Same/better	22 (39.3)	210 (57.9)		

^†^IQR = interquartile range

*Refers to last six months

^Ψ^Refers to *p* values derived from Fisher’s exact tests. All other *p *values refer to those derived from Pearson’s Chi-squared test (for categorical variables) or Mann–Whitney U test (for continuous variables)

φOdds ratio could not be calculated due to a cell count of zero

**Table 2:** Multivariable logistic regression analyses of factors associated with having difficulty accessing supervised consumption services or overdose prevention sites during the COVID-19 pandemic among 428 people who use drugs in Vancouver, Canada (2020)

Variable	Adjusted odds ratio (AOR)	95% Confidence interval (CI)	*p *value
*Age*			
(per year increase)	0.96	(0.93–0.99)	0.014
≥ Daily crystal methamphetamine use*			
(yes vs. no)	2.60	(1.28–5.30)	0.009
Injection drug use*			
(yes vs. no)	4.06	(1.38–11.90)	0.011
Participation in addiction treatment*			
(yes vs. no)	2.13	(0.94–4.81)	0.070
Non-fatal overdose*			
(yes vs. no)	2.45	(1.24–4.85)	0.010
Unstable housing*			
(yes vs. no)	2.14	(1.08–4.23)	0.029
*Perceived drug quality since* *COVID-19 pandemic*			
(Worse vs. same/better)	1.76	(0.94–3.27)	0.077

^*^Refers to last six months

Among the 58 participants who reported experiencing difficulty accessing SCS, the most commonly reported reasons for experiencing such difficulty were: COVID-19-related site closure or shortened hours (*n* = 24; 42.9%) and having to wait too long to use a site (*n* = 22; 39.3%). Other reported reasons include: site is too hectic (*n* = 8; 14.3%), fear of getting COVID-19 (*n* = 5; 8.9%), and other (*n* = 4; 7.1%).

## Discussion

In this study, we found that approximately one in seven participants in a community-recruited sample of 428 people who use drugs in Vancouver, Canada, interviewed between July and November 2020 reported experiencing difficulty accessing (i.e. having tried but been unable to access) SCS, including federally sanctioned SCS and/or OPS, during the COVID-19 pandemic. In adjusted analyses, participants who were younger, engaged in high-intensity methamphetamine use, recently injected drugs, recently experienced non-fatal overdose, or recently experienced housing instability were more likely to experience difficulty accessing SCS. Among people who use drugs who reported having difficulty accessing SCS, the most commonly cited reasons for experiencing such difficulty included: reduced site hours or closures; long wait times to use a site; and a site being too hectic.

To our knowledge, this is the first quantitative study to characterize difficulty accessing SCS during the COVID-19 pandemic among people who use drugs. We found that a significant minority of people who use drugs experienced difficulty accessing SCS. Difficulty accessing SCS was associated with several notable markers of structural vulnerability and drug-related risk including unstable housing, non-fatal overdose, active injection drug use, and daily crystal methamphetamine use. This is concerning given that many of these are characteristics of the primary target client population of SCS [32, 38]. Further, studies conducted prior to the emergence of COVID-19 have found SCS to effectively engage such higher-risk subpopulations of people who use drugs, raising questions about potential inequitable impacts of COVID-19 response measures [6, 31, 32, 38]. In the context of the current overdose crisis, it is particularly concerning that people who use drugs who reported recently experiencing a non-fatal overdose were more likely to experience difficulty accessing SCS, especially given that non-fatal overdose is a strong predictor of future fatal overdose [39, 40] and that people who have recently overdosed may seek to use SCS as a strategy to reduce risk of overdose-related harm [41]. This association suggests potential missed opportunities to engage this population in an intervention that has been found to be highly effective in mitigating overdose-related morbidity and mortality [5–7]. Moreover, studies conducted in the pre-COVID-19 era have found that experiencing barriers in accessing SCS may prompt people who use drugs to engage in unsafe injection practices that increase risk of overdose-related harms, including using in public and using alone [42, 43]. Similarly concerning is our finding that people who recently injected drugs were more likely to experience barriers in accessing SCS, particularly given that this population is known to be at heightened risk of HIV and other viral and bacterial infections, and that SCS afford protection against such harms [6, 8, 10, 11], which is a key reason cited for using these services among people who inject drugs [41].

We found that the most common reason for experiencing barriers in accessing SCS during the COVID-19 era was site closures/reduced hours due to COVID-19 response measures. This suggests that COVID-19-related restrictions on SCS operations during the early months of the pandemic played a role in disrupting access to and coverage of these services among people who use drugs in this setting. Many people who use drugs also reported that programmatic issues during times when SCS were open played a role in impeding access, including long wait times and SCS being too hectic, which were the second and third most common reasons for having difficulty accessing SCS. Such programmatic issues could be due in part to efforts to restrict SCS capacity to facilitate physical distancing, although it should be noted that previous studies undertaken in Vancouver prior to the emergence of COVID-19 documented a pre-existing unmet need for SCS among local people who use drugs [42, 44]. Given that many COVID-19-related restrictions on SCS operations that were in place in the early months of the pandemic (e.g. closures; reduced capacity) have since been lifted [45, 46], future research should be undertaken to assess if and the extent to which difficulty accessing such services among people who use drugs may have decreased over time.

Our findings also point to the need for additional strategies to support access to SCS as part of current and future pandemic and overdose crisis response efforts. One such potential strategy could be to establish additional temporary SCS such as OPS and consider integrating these into existing service settings or utilizing makeshift spaces (e.g. trailers). This has been shown to be a feasible and effective approach in facilitating the rapid scale-up of SCS in response to a public health emergency [7, 46, 47]. Employing flexible models of SCS may also help to address gaps in service coverage. For example, beginning in BC in 2020, episodic overdose prevention services (e-OPS) allow health and social service providers to supervise drug consumption in community and outreach settings outside of established SCS [48]. Possible e-OPS settings include acute care facilities, community isolation shelters, and supportive housing settings [48]. Expanding e-OPS shows promise as an effective approach to reduce harms associated with illicit drug use during a concurrent pandemic by providing low-barrier SCS within spaces that are already engaging people who use drugs, thus increasing accessibility while mitigating risk of COVID-19 transmission [40]. Further, alley patrol services have been expanded in some settings, including in Vancouver, as part of efforts to address service gaps stemming from COVID-19-related SCS closures and disruptions [49–51]. These services provide sterile drug use supplies and naloxone to people who use drugs in public settings, while also responding to overdoses and recovering publicly discarded used drug use equipment. However, compared to SCS, alley patrol services may be limited in their ability to mitigate harms given that these services do not provide secure, hygienic physical spaces in which drug consumption can be supervised [51]. Future studies should seek to explore the effectiveness of the aforementioned strategies in the context of the intersecting overdose crisis and COVID-19 pandemic.

This study has several limitations. First, the cross-sectional study design limits our ability to infer temporality of observed associations between explanatory variables and difficulty accessing SCS during the COVID-19 pandemic. The study also relied on self-reported data, and therefore, our findings are susceptible to reporting biases such as social desirability and recall biases. Additionally, inhalation or smoking of substances was not assessed in the study questionnaire. Given that most SCS in Vancouver do not currently accommodate drug inhalation, future studies should seek to assess the extent to which this factor may act as a barrier to engagement with SCS. The VIDUS and ACCESS cohorts are community-recruited, non-randomized samples, which limits the generalizability of these findings to people who use drugs in Vancouver and other settings. It is also possible that selection bias was introduced due to remote data collection. Specifically, people who use drugs who were most marginalized, including those who lacked access to a phone or internet, may have been less likely to have been included in the study sample, and thus, the observed prevalence of the outcome is likely to be an underestimate. An additional limitation is that, for study participants interviewed between July and mid-September, the past-six-month recall period for the outcome variable of difficulty accessing SCS could refer to experiences prior to the emergence of the COVID-19 pandemic. Given that these participants may have been less likely to experience barriers in access, our observed prevalence of the outcome could be an underestimate of the prevalence of difficulty accessing SCS during the COVID-19 pandemic.

## Conclusions

In summary, we found that approximately one in seven people who use drugs in Vancouver experienced difficulty accessing SCS during the COVID-19 pandemic. Those that reported difficulty accessing such services were more likely to contend with markers of structural vulnerability and drug-related risk. These findings point to the need for further strategies to support access to SCS among people who use drugs as part of the response to the concurrent overdose and COVID-19 crises.

## Data Availability

The data used for this study are not publicly available. For further information on the data and materials used in this study, please contact the corresponding author.

## Abbreviations

ACCESS: AIDS Care Cohort to evaluate Exposure to Survival Services
AIC: Akaike information criterion
AOR: Adjusted odds ratio
BC: British Columbia
CI: Confidence interval
e-OPS: Episodic overdose prevention site
OPS: Overdose prevention site
OR: Odds ratio
SCS: Supervised consumption services
VIDUS: Vancouver Injection Drug Users Study
